# Flaxseed Ethanol Extracts’ Antitumor, Antioxidant, and Anti-Inflammatory Potential

**DOI:** 10.3390/antiox11050892

**Published:** 2022-04-30

**Authors:** Elisabeta Ioana Chera, Raluca Maria Pop, Marcel Pârvu, Olga Sorițău, Ana Uifălean, Florinela Adriana Cătoi, Andra Cecan, Andrada Gabriela Negoescu, Patriciu Achimaș-Cadariu, Alina Elena Pârvu

**Affiliations:** 1 Department of Pathophysiology, Iuliu Hațieganu University of Medicine and Pharmacy, 400012 Cluj-Napoca, Romania; elisabeta.chera@yahoo.com (E.I.C.); uifaleanana@gmail.com (A.U.); florinela12@yahoo.com (F.A.C.); andra.cecan@umfcluj.com (A.C.); parvualinaelena@umfcluj.ro (A.E.P.); 2 Department of Pharmacology, Toxicology and Clinical Pharmacology, Iuliu Haţieganu University of Medicine and Pharmacy, 400337 Cluj-Napoca, Romania; 3 Faculty of Biology and Geology, Babeș-Bolyai University, 400015 Cluj-Napoca, Romania; marcel.parvu@ubbcluj.ro; 4 Department of Hematology, Ion Chiricuță Institute of Oncology, 400015 Cluj-Napoca, Romania; soritau@iocn.ro; 5 Department of Pathology, University of Agricultural Sciences and Veterinary Medicine, 400372 Cluj-Napoca, Romania; andrada-negoescu@student.usamvcluj.ro; 6 Department of Oncology, University of Medicine and Pharmacy Iuliu Hațieganu, 400012 Cluj-Napoca, Romania; pachimas@umfcluj.ro

**Keywords:** antitumoral, Ehrlich ascites carcinoma, flaxseed, antioxidant, anti-inflammatory

## Abstract

The antitumoral, antioxidant, and anti-inflammatory effects of flaxseed ethanol extract was screened. Phytochemical analysis was performed by measuring the total phenolic content and by HPLC-DAD-ESI MS. In vitro antiproliferative activity was appreciated by MMT test of four adenocarcinomas and two normal cell lines. In vitro, antioxidant activity was evaluated by DPPH, FRAP, H_2_O_2_, and NO scavenging tests. The in vivo growth inhibitory activity against Ehrlich ascites carcinoma (EAC) in female BALB/c mice was determined using the trypan blue test. In EAC mice serum and ascites total oxidative status, total antioxidant reactivity, oxidative stress index, malondialdehyde, total thiols, total nitrites, 3-nitrotyrosine, and NFkB were measured. The phytochemical analysis found an significant content of phenols, with lignans having the highest concentration. The extract had an significant in vitro antioxidant effect and different inhibitory effects on different cell lines. After treatment of EAC mice with flaxseeds extract, body weight, ascites volume and viable tumour cell count, serum and ascites oxidative stress, and inflammatory markers decreased significantly. The ethanol flaxseeds extract has potential antiproliferative activity against some ovary and endometrial malignant cells and EAC. This effect can be attributed to the phenols content, and its antioxidant and anti-inflammatory activity.

## 1. Introduction

Cancer is the leading mortality cause worldwide. Each year, nine million people are diagnosed and around five million die [1]. The current antitumor treatments, chemotherapy, radiotherapy, targeted therapy, hemotherapy, surgery, and immunotherapy, have many limitations such as secondary effects due to organ toxicity, low selectivity for neoplastic cells, injuring normal cells as well; high cost; and unavailability in the countries with limited resources [2,3,4]. Therefore, there is a continuous search for new and effective drugs.

The tumour microenvironment includes not only malignant cells, but also non-malignant cells such as endothelial cells, fibroblasts, macrophages, neutrophils, and others. Initially, these non-malignant cells contribute to tumour suppression. Then, the tumour initiates complex inflammatory and immune responses, inflammatory cells are recruited, and when tissue homeostasis is lost the tumour microenvironment changes to become a tumour promoter [5]. Several studies have shown that tumour growth can trigger antioxidant deficiencies and ROS excess [6]. On the other hand, neutrophils and macrophages produce ROS as a mechanism for tumour cell destruction, high levels of ROS triggering cell cycle arrest, apoptosis, and/or senescence. Finally, the interactions between malignant and non-malignant cells trigger chronic proliferative and disseminative signals [5]. Oxidative stress and inflammation are closely correlated pathophysiological processes, one of which is induced by the other. Thus, a recent therapeutic option in cancer is to look for substances that specifically target both inflammation and oxidative stress [7].

The inverse association between diets rich in vegetables and fruits and a lower risk of cancer [3] directed studies upon medicinal plants. Phytotherapy effects in cancer treatment are attributed to the phytochemicals present in plants [3]. In humans, some phytochemicals (e.g., bleomycin, vincristine, and vinblastine) are already used for the treatment of several cancers [2,3]. Many studies revealed that phenolic compounds of the plants’ products may block or suppress cancer, either due to antioxidant activities or by interfering with the cell cycle, apoptosis, and metastasis [1]. Moreover, some phytochemicals may also act as pro-oxidants on cancer cells, leading to cancer cell growth inhibition [2]. Thus, continuous efforts need to be exerted to find new and effective plants with antineoplastic activities and lower toxicity and side effects.

Flaxseed (*Linum usitatissimum* L.), the seed from the flax plant belonging to the *Linaceae* family, is known mostly as a “functional food” due to its nutritional quality. At the same time, it decreases the risk of cancer, atherosclerosis, cardiovascular diseases, insulin-dependent diabetes mellitus, and hyperlipoproteinemia, and reduces menopausal symptoms [8,9,10,11]. Flaxseed health benefits were associated with some of its biologically active phytochemicals [12]. It is a rich source of dietary fibre, alpha-linoleic acid (ALA), and the richest source of plant lignans. ALA has an anti-inflammatory effect by inhibiting the pro-inflammatory prostaglandin synthesis, and lignans have phytoestrogenic and antioxidant activities [13,14,15]. Phytoestrogens are naturally nonsteroidal phenolic plant compounds that can act as oestrogen receptor agonists or antagonists [16], as well as antiangiogenic, antimetastatic, and epigenetic substances. Furthermore, phytoestrogens can reverse multidrug resistance [17].

This study aimed to evaluate flaxseed ethanol extract anticancer activity because only the flaxseed, flaxseed oil, or some compounds from the flaxseed were tested. Moreover, because it was demonstrated that flaxseed oil had variable activity on cell proliferation [8], we wanted to find if flaxseed ethanol extract also has different effects on malignant and nonmalignant cells. Considering ovarian and endometrial adenocarcinomas are the most lethal gynaecologic malignancy in women, we evaluated flaxseed extract effects on one human endometrial adenocarcinoma and three human ovarian adenocarcinoma cell lines. To find if both anti-inflammatory and antioxidative effects are involved in flaxseed ethanol extract antiproliferative activity, we tested the flaxseed extract in vivo on an Ehrlich ascites carcinoma (EAC) model.

## 2. Materials and Methods

### 2.1. Chemicals and Cell Cultures

Methanol, Folin–Ciocâlteu reagent, vanadium chloride (III) (VCl3), sulfanylamide (SULF), N- (1-Naphthyl) ethylenediamine dihydrochloric acid (NEDD), ferrous ammonium sulfate, xylenol orange [ocresosulfonphthalein-3,3-bis (sodium methyliminodiacetate)], orthodianisidinedihydrochloric acid (3-3′-dimethoxybenzidine), hydrogen peroxide (H_2_O_2_), sulfuric acid, hydrochloric acid, glycerol, ethylenediaminetetra-acetic acid, sodium dodecal, sulfate butylated hydroxytoluene, thiobarbituric acid, 1,1,3,3-tetraethoxypropane, 2,4-dinitrophenylhydrazine (DNPH), 5,5′-dithionitrobis 2-nitrobenzoic acid (DTNB), 1,1-diphenyl-2-picrilhydrazyl (DPPH), and o-phthalaldehyde were purchased from Merck (Darmstadt, Germany); Trolox (6-hydroxy-2,5,7,8-tetramethylchroman-2-carboxylic acid) was purchased from Alfa-Aesar (Karlsruhe, Germany). Phenolic compounds were provided from Sigma (St. Louis, MO, USA). All chemicals were of analytical grade. The 3-nitrotyrosineELISA kit was purchased from ABNOVA (KA0445-ABNOVA EMBLEM, Heidelberg, Germany), and the NfkB ELISA kit from Biothec (EU2560- Fine Biothec, Wuhan, China).

Human ovarian adenocarcinoma cells (NIH:OVCAR-3) (from Cell Line Service, Germany), A2780 ovary adenocarcinoma cell lines, and A2780cis ovary adenocarcinoma cell line resistant to cysplatin acquired from the European Collection of Authenticated Cell Cultures (ECACC) through Sigma-Aldrich (St. Louis, MO, USA). Endometrial adenocarcinoma cell line Ishikawa was obtained from American Type Culture Collection (ATCC). Human normal fibroblast cell line BJ HEP (code CRL-2522), purchased from American Type Culture Collection (ATCC). Human umbilical vein endothelial cells (HUVEC) were obtained from the European Collection of Cell Cultures (ECACC, Porton Down, Salisbury, UK). Dulbecco’s Modified Eagle Environment (DMEM), fetal calf serum (FCS) and resazurine sodium salt were purchased from Sigma Aldrich (Munich, Germany). The phosphate-buffered solution (PBS) and the mixture of penicillin/streptomycin and trypsin-EDTA antibiotics were purchased from Gibco (Karlsruhe, Germany).

### 2.2. Plant Extract Preparation

Flaxseeds (Sanovita SRL, Pausesti—Maglasi, Valcea, Romania) were ground in a coffee grinder (Argis, RC-21, Electroarges SA, Curtea de Arges, Romania) for 5 min, and then screened through a 200 μm Retsch sieve. For flaxseeds ethanolic extract (FLAX) preparation, fifty grams of ground material were extracted with 70% ethanol by using the modified Squibb repercolation method, in the Mycology Laboratory of Babes-Bolyai University, Cluj-Napoca [18]. Briefly, three successive applications of the same menstruum were repercolated to the flaxseed material, and the final extract was 1:1.2 g/mL (w:v).

### 2.3. Phytochemical Analysis

#### 2.3.1. Total Polyphenols Content

The total polyphenol content (TPC) of the FLAX was measured using the Folin–Ciocâlteu method with some modifications. The absorbance was measured at 760 nm with a JASCO UV-VIS spectrophotometer, and results were expressed as gallic acid equivalents (mg GAE/g d.w. plant material). The assay was performed in triplicate [19].

#### 2.3.2. Total Flavonoids Content

The flavonoids was determined according to the methods described previously [20]. Briefly, 1 mL of FLAX was firstly mixed with 0.3 mL of NaNO_2_ 5%, then with 0.3 mL AlCl_3_ 10%, and finally with 2 mL of 1M NaOH solution. Then, the mixture was brought to a final volume of 10 mL with distilled water. After incubation (15 min), the absorption was recorded at 510 nm using a Jasco v530 Spectrophotometer. Quercetin was used for the calibration curve (r^2^ = 0.9914). Total flavonoids were expressed as mg quercetin equivalents (mg QE/100g d.w. plant material).

#### 2.3.3. Identification and Quantification of Polyphenolic Compounds by HPLC-DAD-ESI MS

The FLAX was characterized using an high performance liquid chromatography (Agilent 1200 HPLC) with DAD detection which was coupled to a single quadrupole mass spectrometer (Agilent 6110 MS). Separation of compounds was performed using an XDB C18 Eclipse column (4.6 × 150 mm, particle size 5 μm) (Agilent Technologies, Santa Clara, CA, USA) at room temperature. The phenolic compounds of the FLAX were determined as previously described with some modifications [20]. A combination of mobile phase A, which consisted in 0.1% acetic acid/acetonitrile (99:1) in distilled water (*v*/*v*), and mobile phase B, which consisted in 0.1% acetic acid in acetonitrile (*v*/*v*), were used following a gradient program. The program started with 95% A (0–2 min), followed by its decrease as follows: 95–60% A (2–18 min), 60–10% A (18–20 min), and 10% A (20–24 min). After 24 min, the percentage of mobile phase A was increased to 95% in one minute and kept at 95% for another 5 min [20]. A flow rate of 0.5 mL/min was used. The absorbance spectrum was registered at 280 nm specifically for phenolic acids and at 340 nm specifically for flavonoids. Further, the separated compounds were directed towards the MS. Compounds ionization was performed using the ESI source in the (+) mode. The temperature was set at 350 °C, the capillary voltage at 3000 V, and the nitrogen flow at 8 L/min. Compounds were scanned between 100–1000 *m*/*z* interval. Data analysis was performed using the Agilent ChemStation Software (Rev B.04.02 SP1, Palo Alto, CA, USA). The identification of phenolic compounds was achieved using the data obtained from their UV‐visible spectra, retention time, co-chromatography with standards (when available), mass spectra, and literature data. Before LC analysis, the lyophilized extract was dissolved in MeOH. The quantity was expressed as μg/mL gallic acid equivalents for hydroxybenzoic acids, as μg/mL caffeic acid equivalents for hydroxycinnamic acids, and as μg/mL secoisolaricirisenol equivalents for lignans.

#### 2.3.4. Identification and Quantification of Polyphenolic Compounds by FTIR

The FTIR spectra of FLAX was recorded using a Shimatzu IR Prestige- 21 FTIR spectrometer. The apparatus was equipped with attenuated total reflectance (ATR) and a Zinc Selenide (ZnSe) Composite as internal reflection accessory. The extracts were pipetted directly on the crystal and were further evaporated. The spectra was then registered between 4000–650 cm^−1^. Air spectrum was used as background.

### 2.4. In Vitro Antioxidant Activity Analysis

DPPH radical scavenging activity of FLAX was measured as previously described [7]. Briefly, 3 mL of extract, 1 mL DPPH, and 0.1 mM methanol solution were added. After 30 min at room temperature and in the dark, absorbance was measured at 517 nm against a blank, and the percentage of radical scavenging activity (AA%) was calculated by the formula: AA% = [(A control − A sample)/A control] × 100. A Trolox standard solutions calibration curve (0.5–5 μg/mL) was used, and AA% was converted to µg TE/g d.w. plant material [18].

The reduction capacity of the FLAX was measured with the FRAP (ferric reducing antioxidant power) assay as previously described [21]. Briefly, 3.4 μL FRAP reagent and 100 μL FLAX sample were added and mixed thoroughly. After 30 min, the absorbance was taken at 593 nm, and the results were expressed as IC_50_ in TE (µg TE/g d.w. plant material).

The ability of the FLAX to scavenge hydrogen peroxide was determined as previously described [22]. Briefly, FLAX in distilled water was added to H_2_O_2_ solution and after 10 min absorbance was taken at 230 nm against a blank solution of phosphate buffer. The percentage of H_2_O_2_ scavenging was calculated as follows: % scavenged H_2_O_2_ = (A control − A sample/A control) × 100 [23]. The results were expressed as IC_50_ in TE (mg TE/g d.w. plant material).

The Nitric Oxide Radical Scavenging Assay was performed as previously described [24]. Nitric oxide (NO) was generated from sodium nitroprusside, and it was measured by the Griess reagent. Briefly, FlLAX (0.5 mL) was added to an SNP solution (2 mL SNP and 0.5 mL PBS, pH 7.4), and then incubated for 2.5 h at 25 °C; 0.5 mL of this mixture was added to 1 mL sulphanilic acid; after 5 min 1 mL Naphthylethylene-diamine-dihydrochloride was added to the mixture; the final mixture was vortexed and incubated for 30 min. The absorbance was read at 546 nm, and the percentage of inhibition was calculated as follows: % inhibition = (A blank − A sample/A blank) × 100. Results were expressed as IC_50_ in TE (mg TE/g d.w. plant material) [24].

All antioxidant assays were performed in triplicate.

### 2.5. Cell Cultures and Cytotoxicity Test

#### 2.5.1. Cell Lines

NIH:OVCAR-3 cell line was grown in RPMI-1640 culture medium (Sigma-Aldrich, Germany), supplemented with 2 mM L-glutamine, 100 U/mL penicillin/100 µg/mL streptomycin (Sigma-Aldrich), 15% fetal bovine serum (FBS) (Sigma-Aldrich, Germany), and 0.01 mg/mL bovine insulin (Sigma-Aldrich). A2780 and A2780 Cis cell lines were cultivated in RPMI 1640 medium supplemented with 10% Fetal Bovine Serum (Sigma Aldrich), 2 mM L-glutamine, 1% antibiotics (penicillin + streptomycin) (all reagents from Sigma Aldrich) and with 1 µM cisplatinum in the case of A2780 Cis cell line. Ishikawa cell line was cultivated in Eagle’s Minimal Essential Medium (MEM) supplemented with 10% FBS, 2 mM L-glutamin, 1 mM sodium pyruvate, 1% NEA, and 1% antibiotics [25]. BJ HEP cell line was cultivated in Eagle’s Minimal Essential Medium (MEM), supplemented with 10% Fetal Bovine Serum (Sigma Aldrich), 2 mM L-glutamine, 1% antibiotics (penicillin + streptomycin), and 1% Non-Essential Aminoacids (NEA) Solution (Sigma Aldrich) [26]. HUVEC cell line was cultivated in RPMI medium, with 10% Fetal Bovine Serum (Sigma Aldrich), 2 mM L-glutamine, and 1% antibiotics (penicillin + streptomycin) (all reagents from Sigma Aldrich). Cell cultures in the 12th passage were used [27].

#### 2.5.2. MTT Viability Assay

After performing several passages, the cells were trypsinized with 0.25% trypsin + EDTA, after 3 washes with phosphate-buffered saline (PBS). Trypsin was inactivated with a complete medium containing 10% FBS and cells were centrifuged at 1100 rpm for 5 min. The cell suspension was counted with a cell counter EVE^TM^ and cell viability was assessed with trypan blue. The number of cells was adjusted and the cells were seeded in 96-well plates at a density of 1 × 10^4^ in 200 μL complete medium. Each test was performed in triplicate. For 24 h, the cells were allowed to adhere, after which the flaxseed extract was added in decreasing concentrations.

The MTT viability test was performed to determine the cytotoxicity and subsequent working doses. Cell viability was assessed after 24 h of exposure to FLAX. The method consisted in removing the culture medium from the cells and adding 100 µL MTT solution (1 mg/mL)/well. After one hour of incubation at 37 °C in the dark, the MTT solution was extracted from the wells and 150 μL of DMSO/well was added to solubilize the formazan crystals in the cells. Optical density readings were performed at 492 nm using a BioTek Synergy 2 microplate reader (Winooski, VT, USA). Using a GraphPad Prism 5 statistical program, the results were analysed using one-way ANOVA with Dunnett’s Multiple Comparison Test statistical analysis method to compare treated cells to untreated control samples. Significant values were set for a *p* < 0.05 (*).

All experiments were performed in triplicate.

### 2.6. Experimental Design

#### 2.6.1. Animal Subjects

Female BALB/c mice aged 6–7 weeks, with a mean weight of 17.9 (±0.34) g, were used to evaluate the effect of FLAX on the EAC. The animals were housed in polypropylene cages, with free access to water and standard pellet food, and in a controlled laboratory environment (12 h light/dark cycles, at an ambient temperature of 21 °C). The experiments were performed according to the national and international guidelines for animal care and use, followed the Helsinki Declaration on animal studies and received ethical approval from both the “Iuliu Hațieganu” University of Medicine and Pharmacy Cluj-Napoca and the Romanian National Sanitary Veterinary and Food Safety Authority (nr. 168/6 June 2019). All experiments were conducted in triplicate.

#### 2.6.2. Experimental Protocol

The Ehrlich ascites carcinoma (EAC) model was used to evaluate the in vivo antitumor activity of FLAX. The animals were divided into three groups (n = 10) as follows: negative control (CONTROL) received saline (5 mL/100g b.w. 0.9% NaCl *w*/*v* p.o); EAC group received 4 × 10^6^ EAC cells/mice in the peritoneal cavity with a volume of 0.2 mL in PBS; EAC with FLAX treatment group (EAC-FLAX) received 4 × 10^6^ EAC cells/mice in the peritoneal cavity with a volume of 0.2 mL in PBS, and from day 8 FLAX was administrated by gavage (5ml FLAX/100g b.w. p.o., equivalent to 4.16 g dry plant material/kg b.w.) for 10 consecutive days [6,28]. The dose was calculated knowing that daily mice food intake is about 4.4 ± 0.1 g/day/10 g b.w., and recommended flaxseed flour dose was 10% from the daily food intake [29]. We did not perform in vivo toxicity tests because toxicity has never been reported [30].

On day 1 and 17, body weight (BW) was measured (g). On the 18th day, after a 6 h fast, all animals were anaesthetized with ketamine hydrochloride (100 mg/kg, i.m.) and xylazine hydrochloride (16 mg/kg, i.p.), and peripheral blood samples were harvested from the retro-orbital plexus. Then, the animals were euthanized by cervical dislocation, ascites fluid was collected from the peritoneal cavity, and volume was measured (mL).

#### 2.6.3. EAC Tumour Cell Viability

Trypan blue assay was used to evaluate the EAC tumour cell viability in ascites fluid. After tumour induction and treatment plan, EAC cells were collected from the peritoneal cavity of experimental mice, suspended in saline, mixed with trypan blue dye (0.4%), and counted in a Neubauer hemocytometer as previously described [23,24]. The cells with injured membranes were stained, and cells with intact membranes were not stained.

### 2.7. In Vivo Oxidative Stress and Anti-Inflammatory Markers

Oxidative stress was assessed by measuring total antioxidant reactivity (TAR), total oxidative status (TOS), oxidative stress index (OSI, total thiols (SH), and malondialdehyde (MDA).

TAR was measured with a colourimetric assay [31]. Briefly, via the Fenton reaction, hydroxyl radical was produced, and the rate of the reactions was monitored by measuring the absorbance of coloured dianisidyl radicals at 444 nm. Antioxidants from the FLAX sample suppressed the colour formation according to their concentrations. This assay was calibrated using TE, and results are expressed as mmol TE/L.

The total oxidative status (TOS) is a colourimetric assay based on the oxidation of ferrous ions to ferric ions by the oxidants from the FLAX in an acidic medium. Then, ferric ion makes a coloured complex with xylenol orange. Sample absorbance was measured at 560 nm [32]. A standard curve of H_2_O_2_ was used, and assay results were expressed in μmol H_2_O_2_ equiv/L. The ratio of the TOS to the TAR represents the oxidative stress index (OSI), an indicator of the degree of oxidative stress [33].

Total thiols (SH) were estimated using modified Ellman’s reagent [34]. Briefly, 0.6 mL of 20 mM Tris-HCl buffer pH 8.2 was added to 0.2 mL FLAX, followed by the addition of 0.04 mL of 10 mM DTNB in absolute methanol and 3.16 mL of absolute methanol. After 15 min at room temperature, supernatant absorbance was measured at 412 nm. A standard curve of glutathione (GSH), ranging from 0.25 to 2 mM GSH, was used, and serum SH concentration was expressed as mM GSH/mL.

Malondialdehyde (MDA), a lipid peroxidation marker, was measured by using thiobarbituric acid, as previously described [35]. After 30 min at 95 °C incubation, sample absorbance was measured at 532 nm. MDA concentration was expressed as nmol/mL of serum.

The anti-inflammatory effect was assessed by measuring total nitrates and nitrates (NOx), 3-nitrotyrosine (3NT) and Nuclear factor-κB (NF-κB) [18,36]. To indirectly determine NO synthesis (NOx), the Griess reaction was used. First serum proteins were removed by extraction with a 3:1 (*v*/*v*) solution of methanol/diethyl ether [33], and nitrates were reduced to nitrites by adding vanadium (III) chloride. Then, Griess reagent was added, and the mixture was incubated for 30 min at 37 °C. Sample absorbance was read at 540 nm, and the concentration of serum NOx was determined using a sodium nitrite-based curve and expressed as nitrite μmol/L [34].

3NT and NF-κB were measured with ELISA kits according to manufacturer instructions.

### 2.8. Statistical Analysis

All results were expressed as mean ± standard deviation (SD) whenever data were normally distributed. Different experimental groups were compared using the one-way ANOVA test and Bonferroni–Holm post hoc test. The correlations analysis was performed with the Pearson test. A *p* < 0.05 was considered statistically significant. The statistical analysis was performed with IBM SPSS Statistics, version 20 (SPSS Inc., Chicago, IL, USA).

## 3. Results

### 3.1. Phytochemical Analysis

**Figure 1:**
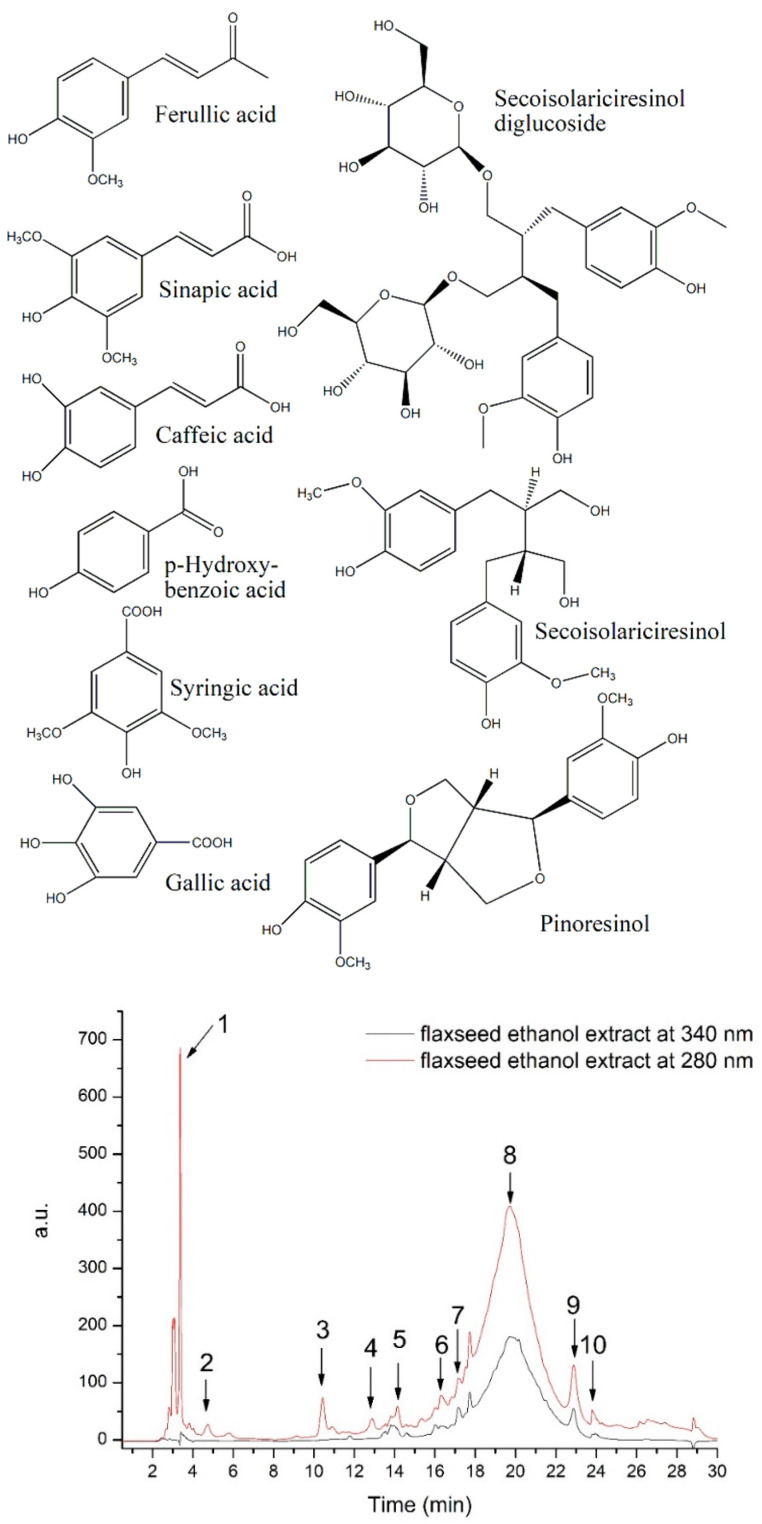
HPLC chromatogram of flaxseed ethanol extract and chemical structure of main identified peaks as listed in Table 1.

The FLAX total polyphenols content (TPC) was 95.18 mg GAE/100 g d.w. plant material, while the total flavonoid content was of 28.61 mg QE/100g d.w. plant material. In our study, HPLC-DAD-ESI MS identified significant concentrations of hydroxybenzoic acids, hydroxycinnamic acids, and lignans (Table 1 and Figure 1).

**Figure 2:**
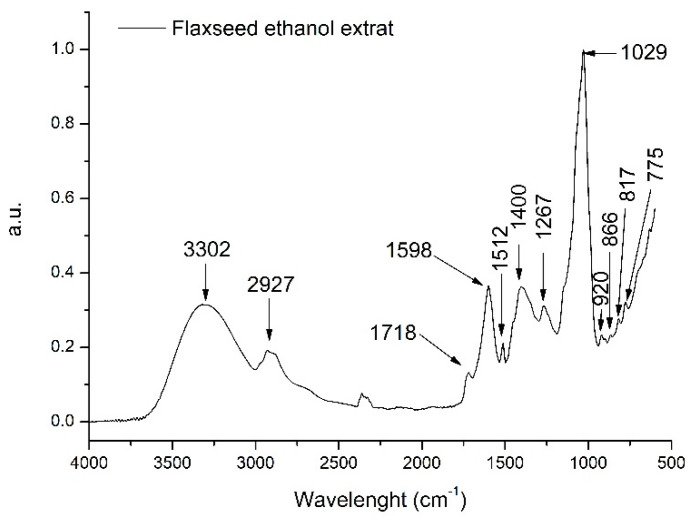
Flaxseed ethanolic general FTIR spectra (3500–500 cm^−1^).

To further identify other compounds extracted from the flaxseed in the ethanolic extract, FTIR analysis was performed. The identification of other extracted compounds can be performed taking into account the absorption spectrum which correlates to the different chemical bonds and functional groups found in the extracted compounds. Figure 2 presents the general FT-IR spectra (3500–500 cm^−1^) of FLAX.

Flaxseed ethanolic extract had 12 major peaks. The broad absorption peak at 3302 cm^−1^ could be assigned to hydroxyl group characteristic to phenolics and phenolic glycosides [37]. The next band at 2927 cm^−1^ can be attributed to a –CH bond in the alkane spectrum [37]. Further, the other major absorption peak at 1029 cm^−1^ could indicate CO stretching vibration from the pyranose ring due to the presence of polysaccharides [38]. The other medium intensity peak at 1598 cm^−1^, found in the interval between 1500–1800 cm^–1^, can be assigned due to the presence of pectic polysaccharides in the FLAX [39]. Further, the interval between 1500–1150 cm^−1^ is characteristic to the CH absorptions bands from phenols and OH deformation vibrations [40]. Thus, the FTIR spectra of the FLAX sample revealed, additionally to phenolics, typical polysaccharide absorption peaks.

### 3.2. In Vitro Antioxidant Activity Analysis

#### 3.2.1. DPPH, FRAP, H_2_O_2_, and NO Analysis

The FRAP IC_50_ and DPPH IC_50_ for FLAX were significantly higher than those of Trolox. IC_50_ of the FLAX H_2_O_2_ scavenging activity was above that of Trolox but with a lower significance. IC_50_ of FLAX NO scavenging activity was significantly smaller than for Trolox (Table 2). The correlation analysis between TPC and in vitro antioxidant tests revealed that DPPH (r 0.8907), FRAP (r 0.9076), and H_2_O_2_ (r 0.8201) were significantly correlated to TPC.

#### 3.2.2. MTT Cytotoxicity Test

**Figure 3:**
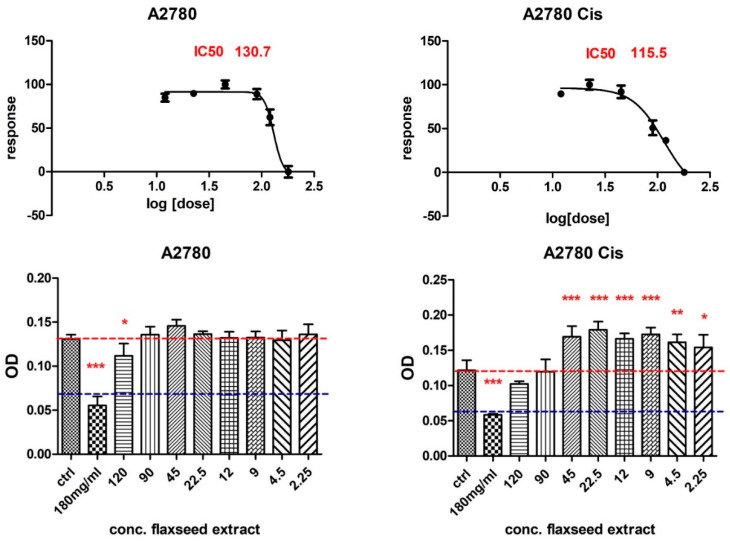
The viability response of ovarian epithelial carcinoma cells A2780 and A2780cis at different concentrations of flaxseed extract (MTT assay), where *p* ≤ 0.05 (*), *p* ≤ 0.01 (**), and *p* ≤ 0.001 (***) as compared with control.

Human epithelial ovarian carcinoma cell line A2780 and its derivative cisplatin-resistant cell line A2780Cis showed different responses to different concentrations of flaxseed extract. At a concentration of 180 mg/mL and 120 mg/mL, a decrease in cell viability was observed for both lines. An interesting aspect was observed in that lower concentrations of extract induced an increase in cell proliferation of the cisplatin-resistant cell line (Figure 3).

**Figure 4:**
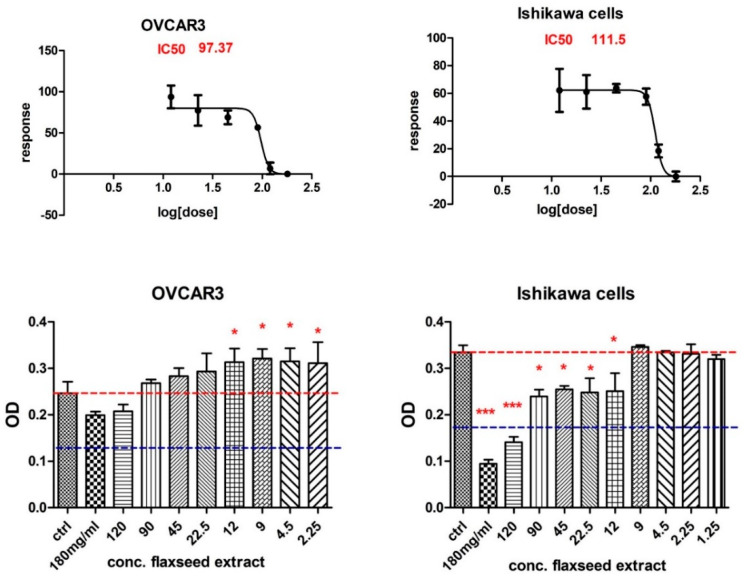
Comparison of the response of viability of ovarian adenocarcinoma cells (NIH:OVCAR-3) and endometrial adenocarcinoma cell line Ishikawa at different concentrations of flaxseed extract (MTT assay), where *p* ≤ 0.05 (*) and *p* ≤ 0.001 (***) as compared with control.

This increase in cell proliferation to the treatment of low-dose flaxseed extract was also observed in the OVCAR3 cell line, which also responded less to doses of 180 and 120 mg/mL of flaxseed extract. In contrast, the Ishikawa endometrial adenocarcinoma cell line is more sensitive, even to lower doses of flaxseed extract (Figure 4).

**Figure 5:**
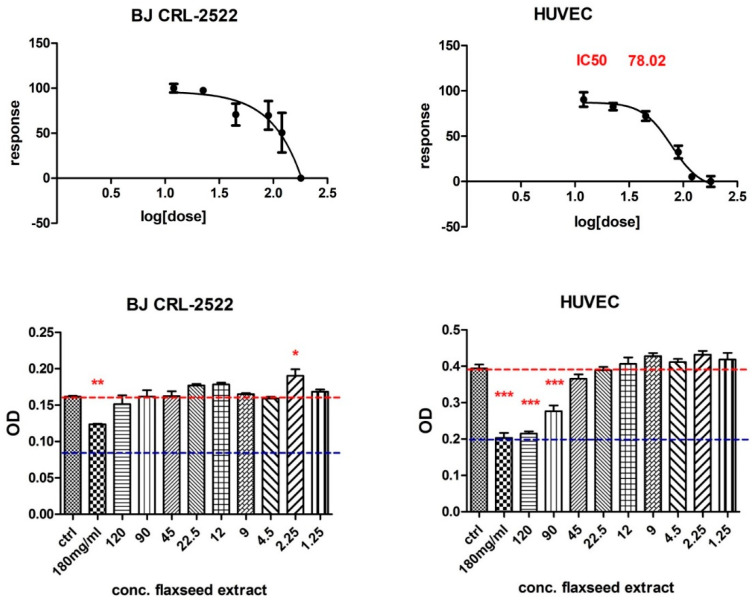
MTT assay results of BJ fibroblasts cells and HUVEC cells treated with different concentrations of flaxseed extract, where *p* ≤ 0.05 (*), *p* ≤ 0.01 (**), and *p* ≤ 0.001 (***) as compared with control.

Human fibroblasts and human endothelial cells from the umbilical vein also showed different behaviour: endothelial cells were found to be more sensitive regarding cytotoxicity, even at lower doses of flaxseed extract (Figure 5).

### 3.3. Flaxseeds Extract Treatment Effects on the Body Weight, Ascites Volume, and Viable/Nonviable Cell Count in EAC-Bearing Mice

The body weight (BW) of the experimental animals was recorded on every alternative day. The animals injected with EAC showed a significant incidence of tumour at the end of the first 8 days, revealed by an increase in the mice’s BW (18.26 ± 4.05%) as compared to CONTROL. After 10 days of treatment, on the 18th day, we found a higher BW gain in the EAC tumour-bearing animals than in EAC- FLAX animals (Table 3).

After 10 days of treatment, ascites fluid was also collected, and a significant reduction in ascites volume was observed in the EAC tumour-bearing animals treated with FLAX (Table 3). FLAX also significantly reduced cell viability in the ascites fluid when compared to the EAC group (Table 3).

### 3.4. In Vivo Antioxidative and Anti-Inflammatory Effect

Oxidative stress was assessed using global and specific markers. In EAC animals, there was a high serum oxidative stress as compared to the CONTROL. In the ECA group, there was no significant change of TAC, a significant decrease in SH, and a significant increase in TOS, OSI, and MDA (Table 4). FLAX treatment caused a reduction in the serum oxidative stress, reflected in a significant decrease in TOS and MDA, plus a significant increase in SH (Table 4).

Ascites’ oxidative stress markers were also measured. FLAX treatment reduced ascites oxidative stress by increasing TAC and SH, which lowered OSI (Table 5).

For the anti-inflammatory activity evaluation, NOx, 3NT, and NF-kB were measured in the serum and ascites fluid. In sera, EAC caused a very significant increase in NOx, 3NT, and NF-kB. FLAX treatment induced a very significant reduction in 3NT and NF-kB, but just a small decrease in NOx S (Table 6). In ascites fluid, FLAX treatment consistently lowered NOx, 3NT, and NF-kB (Table 7).

## 4. Discussion

The results of this study showed that FLAX has important in vitro and in vivo antitumor, antioxidant, and anti-inflammatory activities due to phytochemicals with antioxidant and anti-inflammatory activities.

Traditional medicine used medicinal plants for millennia [41]. In cancer therapy, plant-derived products always served as vital sources, and are also possible sources for new drugs. The basis of precision medicine is to develop targeted drugs that specifically address tumour-related proteins. Phytochemicals represent an excellent source for targeted therapies and can offer a valuable alternative to chemically synthesized drugs and therapeutic antibodies [42]. Flaxseed attracted attention in the field of diet and disease research due to its bioactive phytochemicals with potential health benefits [3].

It is well known that environmental conditions, such as the climate and geographical location, could greatly influence the phytochemical composition of plants [15]. Therefore, it is important to identify the phytochemicals from each plant product [1]. Different solvents are used for the extraction of phytochemicals from flaxseeds. We used ethanol because it has been recommended as one of the most effective solvents [43,44]. The TPC of FLAX was in the same range (98.8 mg/100 g d.w) as those determined in the Polish defatted flaxseed extract [45]. Total flavonoids were 1.2 lower than the median average identified in the flaxseed extracts obtained from four different cultivars grown in Canada [46]. Chromatographic phytochemical analysis of FLAX found very significant quantities of plant lignans, a high content in SECO, and lower quantities of pinoresinol, lariciresinol, and matairesinol. Most phytoestrogens from the diet are inactive glycoside conjugates [16,17,47,48]. After ingestion, these molecules are hydrolyzed to aglycones (primary metabolites), and then intestinal flora transforms aglycones into secondary metabolites that are more similar to estrogens [49]. Due to this metabolic pathway, the response to the phytoestrogens is not the same in different individuals [48]. Although research data indicate the chemopreventive effect of lignans, the mechanism by which phytoestrogenic lignans prevent cancers is still unclear and requires further study [15].

Phytochemical analysis showed that FLAX also contains a significant amount of other phenolic compounds, as others have previously described [13]. Hydroxybenzoic acid was found in high quantities, while gallic acid and syringic acid were in lower quantities. Hydroxybenzoic acid and its derivatives have significant antioxidant activity and inhibit carcinogenesis [50,51]. Gallic acid has been shown to inhibit carcinogenesis and to have pro-oxidant activity in the presence of metal ions in a concentration-dependent manner [52]. Syringic acid has relevant antioxidant effects [51]. In FLAX, hydroxycinnamic acid derivatives such as caffeic acid, sinapic acid, and ferulic acid were in small quantities. Caffeic acid is synthesized by all plant species, and it has antioxidant, anti-inflammatory, and antineoplastic activities [53,54]. Sinapic acid is widespread in plants, and it has good antioxidant, anti-inflammatory, anticancer, and antimutagenic activity [55]; it is a good inhibitor of 3NT formation [56]. Ferulic acid has antioxidant properties by scavenging ROS and activating DNA repair. It also has anti-inflammatory and anticancer effects [57,58]. Taking the phytochemical composition of FLAX together, antioxidant, anti-inflammatory, and antineoplastic activities were expected. Therefore, we further tested the antioxidant effects of FLAX by using in vitro methods.

Our results indicate that FLAX exhibited a higher FRAP than DPPH, H_2_O_2_, and NO scavenging activities. As compared to Trolox, FLAX DPPH, FRAP, and H_2_O_2_ scavenging capacities were better. As the correlation analysis between the in vitro antioxidant test and TPC was very significant, FLAX antioxidant activity can be attributed to phenolic compounds. The results were in agreement with other studies that analysed other species from the *Lamiaceae* family [21].

Further, because introducing plant products with antioxidant activities in the daily diet can be a suitable solution for preventive or therapeutic medicine, we evaluated FLAX antiproliferative activity on some cell lines [47]. Today, evidence strongly supports early dietary flaxseed intake for ovarian cancer prevention, because flaxseed reduces inflammatory prostaglandin E2, oestrogen receptors, toxic oestrogen metabolites, and enzymes expression, all factors involved in ovarian cancer development [9]. Some studies have shown that dietary lignans might lower the oestrogen-dependent aggressiveness in ovarian cancer cells [59]. We tested FLAX effects upon some ovarian and endometrial adenocarcinoma cell lines. The MTT test showed that treatment with FLAX can inhibit the proliferation of cultured malignant cells A2780, A2780cis, and Ishikawa cells in a dose-dependent manner, but it had no significant effect on NIH:OVCAR-3. On the non-malignant cells, FLAX had different toxic effects. Other studies showed that treatment with flaxseed oil or ω-3 fatty acids inhibited the growth of some cancer cells, such as MCF-7 and MDA-MB-231 breast cancer cells, but did not affect non-malignant MCF-10A cells [7]. Therefore, it is clear that FLAX antiproliferative effect depends on the cell type in both categories, malignant and non-malignant. Such results elevate precision medicine to a need, not just a possibility.

To evaluate FLAX in vivo antitumor, antioxidant, and anti-inflammatory effects, we used the EAC mouse model. EAC is an undifferentiated non-invasive carcinoma, with good transplantation capacity and rapid proliferation, a proper model for testing the in vivo effectiveness of drugs in mice [60,61]. Inoculation of EAC cells into mice peritoneum induced ascites caused a significant increase in the BW and viable tumour cell count [4]. The ascites liquid increase is important for EAC because it is the direct nutritional source for tumour cells [6,62]. FLAX treatment for 10 days reduced ascites volume, BW, and viable cells count. These results indicated that FLAX had a toxic effect on EAC, did not reverse EAC, but caused a significant inhibition of tumour growth. This is in line with other studies testing the effect of substances against EAC [5,6] and suggests that FLAX has significant in vivo antitumor activity against EAC in mice.

In ECA animals, we demonstrated that oxidative stress is associated with TOS and OSI increase and TAR reduction. In line with other studies [62], in EAC mice ROS also caused lipid peroxidation, and subsequently MDA increase, a specific biomarker of oxidative stress. FLAX treatment reduced systemic and ascites oxidative stress by lowering ROS and MDA, and by increasing SH antioxidants. These results indicated that FLAX has in vivo antioxidant potential too, not just in vitro.

Cancer’s chronic inflammatory microenvironment is often associated with its development and progression. There is a tumour-extrinsic inflammation caused by cancer risk factors that stimulate malignant progression. Cancer-intrinsic inflammation can be triggered by cancer-induced mutations and can contribute to malignant progression through the recruitment and activation of inflammatory cells. Moreover, both extrinsic and intrinsic inflammations can result in immunosuppression, another pathological consequence that favours tumour progression. Therefore, targeting inflammation represents an actual strategy, both for cancer prevention and treatment. Such drugs can be considered for adjuvants to chemotherapy and radiotherapy too. There are already many plant-derived products with an anti-inflammatory activity that exhibit chemopreventive activities [63]. Therefore, we tested the anti-inflammatory activity of FLAX in EAC model.

Inflammation-activated macrophages generate ROS and nitric oxide (NO). NO is called a ‘double-edged sword’ because it may have dual but reversed effects in cancer and inflammation. In cancer, at low concentration, NO stimulates tumour progression due to the antiapoptotic, pro-growth, and proangiogenic effects, and at high concentrations, NO inhibits tumour progression due to proapoptotic, antiangiogenic, and antiproliferative effects [61,64]. In inflammation, NO can have a proinflammatory effect at high concentrations or an anti-inflammatory effect at lower concentrations [65]. The interaction of NO with ROS can result in reactive nitrogen species (RNS) [5,66], and a nitration reaction of the protein tyrosine leads to 3-nitrotyrosine (3-NT) formation. The nitration of tyrosine residues in proteins may affect their function [23]. Other authors observed an increase in the systemic and local ROS and NO production in the EAC, leading to NOx and 3NT mice elevation [47,61,64]. FLAX treatment significantly decreased NOx and 3NT levels, suggesting that FLAX antitumor mechanism associates a ROS and NO production dependent anti-inflammatory activity, and does not involve NO’s cytotoxicity. When analysing the antioxidant activity of plant extracts by in vitro and in vivo tests, results are not always correlated. That was the case of FLAX effect upon NO; in vitro and in vivo NO’s reduction activities were not correlated; the in vitro NO scavenging activity was modest whilst the in vivo NOx reduction was significant.

The NF-κB signalling is a major regulating pathway of inflammatory responses by regulating cytokines, chemokines, MHC, and adhesion molecules gene expression. In cancer, NF-κB plays an important role not just in tumour-associated inflammation, but also in tumour cell proliferation and tumour formation and progression [17,67]. In the present study we found that, after EAC induction, the expression of NF-κB increased significantly in both serum and ascites. Therefore, NF-κB is a proper target in cancer therapy [68]. The results from anti-inflammatory studies have indicated that many plant extracts and their phytochemicals exert their biological properties by blocking NF-κB signalling pathways [5,17,47,69]. FLAX treatment had an anti-inflammatory effect by reducing NF-κB almost to the CONTROL level, and this effect can be part of the antitumoral activity.

## 5. Conclusions

The present study demonstrated that flaxseed’s ethanol extract has selective antitumor activity on ovarian and endometrial adenocarcinoma cells. This effect can be attributed to the combined effects of the phytochemicals with anticancer, antioxidant, and anti-inflammatory activities it contains, especially the lignans, which were in the higher concentration. The in vivo antitumor effect in EAC was associated with tumour microenvironment changes, probably induced by the anti-inflammatory and antioxidant activities of the flaxseed ethanol extract. Thus, the findings of this study indicated that flaxseed ethanol extract is a promising therapeutic product for some ovarian and endometrial cancers. Future studies have to find more details about the molecular mechanism involved in the antitumor effect of flaxseeds ethanol extract.

## Figures and Tables

**Table 1:** HPLC-DAD-ESI MS compounds identification and quantification in the flaxseed ethanol extract.

No	Retention Time Rt (min)	UV λmax (nm)	[M+H]+ (*m*/*z*)	Tentative Identification	Subclass	Quantity * μg/mL
1	3.37	270	138	Hydroxybenzoic acid	Hydroxybenzoic acid	107.629
2	4.75	280	171	Gallic acid	Hydroxybenzoic acid	18.219
3	10.43	280	199	Syringic acid	Hydroxybenzoic acid	37.432
4	12.87	320	181	Caffeic acid	Hydroxycinnamic acid	13.374
5	14.13	280	687	Secoisolariciresinol- diglucoside (SDG)	Lignan	5.193
6	16.33	320	225	Sinapic acid	Hydroxycinnamic acid	6.543
7	17.16	321	195	Ferulic acid	Hydroxycinnamic acid	5.426
8	19.72	280	360	Secoisolariciresinol (SECO)	Lignan	210.796
9	22.89	280	358	Pinoresinol	Lignan	10.772
10	23.83	280	358	Matairesinol	Lignan	1.569

* quantity expressed as: μg/mL gallic acid equivalent for hydroxybenzoic acids; μg/mL caffeic acid equivalent for hydroxycinnamic acids; μg/mL secoisolaricirisenol equivalent for lignans.

**Table 2:** Flaxseed ethanol extracts antioxidant activity analysis.

Sample	DPPH	FRAP	H_2_O_2_	NO
FLAX IC_50_(µg TE/g d.w. plant material)	39.07 ± 2.84	64.57 ± 12.09	14.33 ± 3.86	57.72 ± 3.58
TROLOX IC_50_(µg /mL)	11.18 ± 1.22	10.21 ± 0.99	10.77 ± 2.43	86.82 ± 12.49
*p*	0.01	0.001	0.05	0.001

DPPH-DPPH free radical scavenging activity; FRAP-ferric reducing antioxidant power; H_2_O_2_ -hydrogen peroxide scavenging capacity; and NO-nitric oxide radical scavenging assay.

**Table 3:** Flaxseeds extract treatment effects on the body weight, ascites volume, and viable/nonviable cell count at the end of the experiment.

Samples	BW Gain (%)	Ascites (mL)	Viable Cell (%)	Non-Viable Cell (%)
CONTROL	11.02 ± 1.57	-	-	-
EAC	32.55 ± 3.29 ###	9.13 ± 1.35	93.68 ± 2.97	6.32 ± 0.94
EAC-FLAX	19.57 ± 4.77 ###	7.40 ± 1.83 *	85.16 ± 3.70 *	14.84 ± 1.08 ***

EAC-Erlichascite carcinoma; EAC-FLAX-EAC treated with flaxseed ethanol extract; * EAC statistical significance vs. CONTROL; # EAC-FLAX statistical significance vs. EAC; * = *p* < 0.5 and ***/### = *p* < 0.001.

**Table 4:** Serum oxidative stress markers at the end of the experiment.

Samples	TAC (mmol TROLOX Equiv./L)	TOS (µM H_2_O_2_ Equiv./L)	OSI	SH (mM GSH/L)	MDA (nM/L)
CONTROL	1.093 ± 0.002	4.20 ± 0.90	3.84 ± 0.63	351.00 ± 59.10	2.64 ± 0.31
EAC	1.088 ± 0.001 #	7.22 ± 1.42 ##	6.73 ± 0.38 ##	360.78 ± 38.35 ##	5.41 ± 0.26 ##
EAC-FLAX	1.089 ± 0.001	5.77 ± 0.86 *	5.29 ± 0.16	563.25 ± 103.22 ***	3.87 ± 0.40 ***

TAR-total antioxidant reactivity; TOS-total oxidative status; OSI-oxidative stress index; SH-total thiols; MDA-malondialdehyde; CONTROL-negative control; EAC-Erlich ascite carcinoma; EAC-FLAX-EAC treated with flaxseed ethanol extract; * EAC statistical significance vs. CONTROL; # EAC-FLAX statistical significance vs. EAC; */# = *p* < 0.5; ## = *p* < 0.01; and *** = *p* < 0.001.

**Table 5:** Ascites oxidative stress markers at the end of the experiment.

Samples	TAC (mmol Trolox Equiv/L)	TOS (µM H_2_O_2_ Equiv/L)	OSI	SH (mM GSH/L)	MDA (nM/L)
CONTROL	1.099 ± 0.001	12.39 ± 1.22	11.42 ± 1.52	410.00 ± 38.09	2.37 ± 0.21
EAC	1.084 ± 0.001 ##	18.90 ± 4.16 ##	17.33 ± 3.52 #	201.67 ± 52.99 ##	3.36 ± 0.36 ##
EAC-FLAX	1.098 ± 0.003	16.70 ± 2.01 *	15.21 ± 2.18	306.50 ± 59.35 **	3.18 ± 0.23 *

TAR-total antioxidant reactivity; TOS-total oxidative status; OSI-oxidative stress index; SH-total thiols; MDA-malondialdehyde; EAC-Erlich ascite carcinoma; EAC-FLAX-EAC treated with flaxseed ethanol extract; * EAC statistical significance vs. CONTROL; # EAC-FLAX statistical significance vs. EAC; */# = *p* < 0.5; **/## = *p* < 0.01.

**Table 6:** Serum anti-inflammatory markers at the end of the experiment.

Samples	NOx (ng/mL)	3NT (ng/mL)	NF-kB (ng/mL)
CONTROL	26.42 ± 3.54	19.84 ± 3.89	1.9 ± 0.15
EAC	39.54 ± 5.21 ##	41.05 ± 10.09 ###	3.70 ± 0.67 ###
EAC-FLAX	31.02 ± 3.38 **	22.16 ± 9.87 **	1.98 ± 0.09 **

NOx-nitrites and nitrates; 3NT-3-nitrothyrosine; NFkB-nuclear factor kB; CONTROL-negative control; EAC-Erlichascite carcinoma; EAC-FLAX-EAC treated with flaxseed ethanol extract; * EAC statistical significance vs. CONTROL; # EAC-FLAX statistical significance vs. EAC; **/## = *p* < 0.01; and ### = *p* < 0.001.

**Table 7:** Ascites anti-inflammatory markers at the end of the experiment.

Samples	NOx (ng/mL)	3NT (ng/mL)	NF-kB (ng/mL)
CONTROL	7.54 ± 0.04	18.18 ± 2.72	2.15 ± 0.17
EAC	17.20 ± 5.52 ###	52.16 ± 8.71 ###	5.44 ± 0.12 ##
EAC-FLAX	9.63 ± 2.85 **	30.53 ± 7.09 **	4.03 ± 0.39 *

Nox—nitrites and nitrates; 3NT-3-nitrothyrosine; NF-kB-nuclear factor kB; CONTROL-negative control; EAC-Erlich ascite carcinoma; EAC-FLAX-EAC treated with flaxseed ethanol extract; * EAC statistical significance vs. CONTROL; # EAC-FLAX statistical significance vs. EAC; * = *p* < 0.5; **/## = *p* < 0.01; and ### = *p* < 0.001.

## Data Availability

The data presented in this study are available on request from the corresponding author.
